# Multifunctional viral protein γ34.5 manipulates nucleolar protein NOP53 for optimal viral replication of HSV-1

**DOI:** 10.1038/s41419-017-0116-2

**Published:** 2018-01-24

**Authors:** Wen Meng, Shi-Chong Han, Cui-Cui Li, Hui-Jun Dong, Xiao-Jia Wang

**Affiliations:** Key Laboratory of Animal Epidemiology of the Ministry of Agriculture, College of Veterinary Medicine, China Agricultural University, 100193 Beijing, China

## Abstract

To ensure efficient virus replication, herpes simplex virus type 1 (HSV-1) encodes several viral proteins to counter host defense response upon infection. Among these proteins, the multifunctional viral protein γ34.5 crucially interferes with or disrupts several antiviral pathways at multiple levels. The current study shows that γ34.5 utilizes nucleolar protein NOP53 to facilitate the dephosphorylation of eukaryotic initiation factor eIF2α for efficient viral translation. Our study shows that: (1) ectopic expression of NOP53 greatly increases the intracellular and extracellular viral yields of HSV-1 (wild strain F) in type I interferon-deficient Vero cells, and more subtly promotes viral replication of γ34.5 deletion mutant virus HSV-1/Δγ34.5. (2) NOP53 is migrated from nuclei in HSV-1/F infected cells, but is redistributed incompletely after infection by either HSV-1/Δγ34.5 or ICP4 deletion mutant virus HSV-1/d120 (replication inadequate). Ectopic expression of γ34.5, consequently, induces cytoplasmic translocation of NOP53 in response to HSV-1/Δγ34.5 infection. (3) Increase of NOP53, in two forms of transient transfection and *in vitro* expression, attenuates the phosphorylation level of eIF2α in HSV-1/F infected cells, but fails to affect eIF2α phosphorylation induced by HSV-1/Δγ34.5 infection. (4) Knockdown of NOP53, which impairs the specific interaction between γ34.5 and protein phosphatase PP1α, disrupts the ability of γ34.5 to maintain HSV-1 virulence. (5) NOP53 knockdown also significantly reduces tissue damage and decreases viral yield in livers of HSV-1 infected mice. Our findings expand the understanding of the underlying mechanism by which viral protein γ34.5 induces NOP53 redistribution; cytoplasmic NOP53 facilitates γ34.5 recruitment of PP1α to dephosphorylate eIF2α, for optimal viral replication. This paper also demonstrates that blocking the specific interaction between γ34.5 and PP1α would be a useful approach for the development of antiviral agents.

## Introduction

Herpes simplex virus type 1 (HSV-1) infection causes a wide spectrum of outcomes and yields a productive lytic infection or establishes a long-term latent infection^1^. HSV-1 infection triggers a rapid induction of cellular defense responses. One of the earliest responses to infection is activation of double-stranded RNA-dependent protein kinase R (PKR). An important function of activated PKR during viral infection is phosphorylation of the eukayotic translation initiation factor eIF2α, resulting in translational arrest and reduction in the global synthesis of viral and cellular proteins^2^. In some cases, viral invasion also induces other host defense responses, including type I interferon (IFN)^3,4^ and autophagy^5^, which in turn affect viral infection of HSV-1.

The important neurovirulence factor γ34.5 of HSV-1 provides an excellent example of how viruses have evolved to modulate a multitude of host defenses with a very limited genome size^6^. Viral protein γ34.5 of HSV-1 wild type F consists of 263 amino acids, and can be divided into three domains: a 160-aa amino-terminal domain, 10 repeats of 3-aa (Ala-Thr-Pro), and a 73-aa carboxyl-terminal domain^7^. Multiple roles of γ34.5 have emerged from the association of γ34.5 with various cellular proteins in targeting different host pathways. For instance, γ34.5 interacts with TANK-binding kinase 1 (TBK1), suppressing production of type I IFN^8,9^. γ34.5 directly interacts with the mammalian autophagy protein Beclin-1 and antagonizes autophagy^10^. Moreover, HSV-1 has evolved an effective strategy through γ34.5 recruiting protein phosphatase PP1α to reverse the eIF2α-mediated translational arrest, to allow for successful viral replication^11–13^. γ34.5 was initially described over two decades ago, but the specific virus-host interactions mediated by this multifunctional protein are still being elucidated.

NOP53 (GLTSCR2/PICT-1) is localized within the well-known 1.4 Mb tumor-suppressive region of chromosome 19q^14^; its expression is down-regulated or eliminated in various tumors^15–17^. Depression of NOP53 sensitizes cells to DNA damage, delays DNA repair, and abolishes G2/M checkpoint activation^18^. Localization of NOP53 is mediated by multiple unique nucleolar localization sequences^19^. Nucleolar NOP53 can translocate to nucleoplasm and stabilize p53 in response to the ribosomal stress^20^. Our previous study showed that NOP53 blocks type I IFN induction and deactivates retinoic acid-inducible gene RIG-I (not TBK1) by negatively regulating it via K63-linked ubiquitination^21^.

Our preliminary results revealed that the ectopic expression of NOP53 greatly increases the viral yields of HSV-1/F in type I IFN-deficient Vero cells, suggesting NOP53 promotes HSV-1 replication in an IFN-independent manner. Considering that NOP53 shares homology with the yeast 60 S ribosomal protein Nop53p, which in yeast acts as an essential ribosome biogenesis factor^22–24^, we designed a series of experiments and found that NOP53 is involved in γ34.5 recruitment of PP1α for the dephosphorylation of eIF2α. This paper demonstrates that viral protein γ34.5 utilizes cellular protein NOP53 for efficient viral replication.

## Results

### NOP53 promotes the production of viral particles and level of viral proteins of HSV-1/F in IFN-deficient Vero cells

**Fig. 1:**
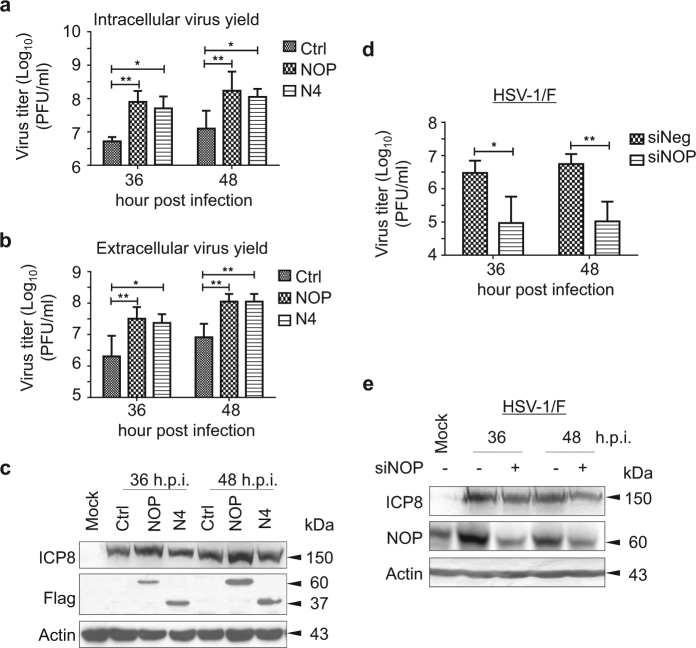
NOP53 promotes the replication of HSV-1/F in Vero cells **a,b** Vero cells seeded in T-25 flask were transiently transfected with control (Ctrl) plasmid, plasmids to express Flag-tagged wt NOP53 (NOP) or truncated N4 (5 μg each) for 36 h. The cells were infected with HSV-1/F at 0.1 MOI, and intracellular viral yields **a** or extracellular viral yields **b** were determined 36 or 48 h postinfection (h.p.i.) by plaque assay. They are presented as log_10_ PFU/ml. These experiments were performed two times with three replicates in each experiment. Values represent means with standard deviations (SD). *p ≤ 0.05; **p ≤ 0.01. **c** Vero cells were transfected with control plasmid, Flag-tagged NOP53, or Flag-tagged N4, and then infected with HSV-1/F at 0.1 MOI. Cell lysates prepared 36 or 48 h.p.i. were then analyzed by Western blotting using antibodies directed against HSV-ICP8 and Flag. Actin served as the loading control. **d** Vero cells seeded in T-25 flask were transfected with specific siRNA targeting NOP53 (siNOP) or negative siRNA (siNeg) (200 pmol each) for 72 h. The cells were then infected with HSV-1/F at 0.1 MOI, and viral yields were determined 36 or 48 h.p.i. by plaque assay. They are presented as log_10_ PFU/ml. Values represent means of triplicates with SD. *p ≤ 0.05; **p ≤ 0.01. **e** Vero cells were transfected with siNOP or siNeg, followed by infection with HSV-1/F at 0.1 MOI. Cell lysates prepared 36 or 48 h.p.i. were then analyzed by Western blotting with the indicated antibodies

In the present study, Vero cells were selected to explore whether NOP53 plays a role in wild-type virus HSV-1/F replication, because the cells do not secrete IFN-α/β when infected by viruses^25^. We ectopically expressed the wild-type (wt) Flag-tagged NOP53 (residues 1 to 478), truncated NOP53-N4 (residues 250 to 478), or negative control and then infected them with HSV-1/F at the MOI of 0.1. As shown in Figs. 1a and 1b, increase of NOP53 and NOP53-N4 resulted in increases of intracellular viral yields by 14.3 and 8.8 fold, respectively, at 36 h post-infection (h.p.i.), and by 13.6 and 8.9 fold, respectively, at 48 h.p.i., in comparison with the negative control infected with HSV-1/F (Fig. 1a). Increase of NOP53 and NOP53-N4 resulted in increase of extracellular viral yields by 14.7 and 10.6 fold, respectively, at 36 h.p.i., and by 12.6 and 12.6 fold, respectively, at 48 h.p.i. (Fig. 1b). We also detected that ectopic expression of the full length NOP53 enhances viral replication, indexed by viral protein HSV-ICP8, while NOP53-N4 appears to enhance viral replication to a level comparable to that achieved with wt NOP53 (Fig. 1c). These preliminary results indicated that the integrity of the C-terminal region N4 is important for NOP53 in support of viral replication. We then found that small interfering RNA (siRNA)-mediated knockdown of NOP53 results in reduction of viral yields by 31.1 and 52.3 fold at 36 and 48 h.p.i., respectively (Fig. 1d). Western blot results also showed that the level of viral protein HSV-ICP8 is down-regulated (Fig. 1e). In addition, over-expression or knockdown of NOP53 did not cause measurable decrease in cell viability (Supplementary figure 1). These results suggest that NOP53 promotes the production of viral particles and increases the level of viral proteins of HSV-1/F in IFN-independent fashion.

### NOP53 improves *de novo* protein synthesis of HSV-1/F

**Fig. 2:**
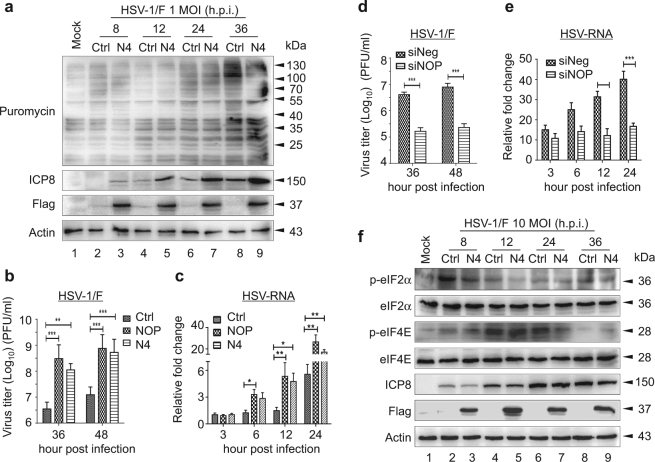
NOP53 improves viral protein synthesis of HSV-1/F **a** HeLa cells transfected with control plasmid (lanes 2, 4, 6, 8) or Flag-tagged N4 (lanes 3, 5, 7, 9) were mock-infected (lane 1) or infected with HSV-1/F at 1 MOI for indicated times (lanes 2–9). Cells were metabolically pulse-chase labeled with puromycin for 1 h prior to harvest. Cell lysates were analyzed by Western blotting with the indicated antibodies. *De novo* protein synthesis was assessed using anti-puromycin antibody 12D10. **b** HeLa cells were transfected with control plasmid, Flag-tagged NOP53, or Flag-tagged N4. The cells were infected with HSV-1/F at 0.1 MOI, and viral yields were determined 36 or 48 h.p.i. by plaque assay. These experiments were performed two times with three replicates in each experiment. Values represent means with SD. **p ≤ 0.01; ***p ≤ 0.001. **c** HeLa cells seeded in 24-well plate were transfected with control plasmid, Flag-tagged NOP53 or Flag-tagged N4 (500 ng each), and then infected with HSV-1/F at 0.1 MOI for the indicated times. Total RNAs were isolated; expression levels of HSV-ICP8 mRNA were quantified by qRT-PCR and normalized with glyceraldehyde-3-phosphate dehydrogenase (GAPDH). These experiments were performed two times with three replicates in each experiment. Values represent means with SD. *p ≤ 0.05; **p ≤ 0.01; ***p ≤ 0.001. **d** HeLa cells were transfected with siNOP or siNeg followed by infection with HSV-1/F at 0.1 MOI, and viral yields were determined 36 or 48 h.p.i. by plaque assay. These experiments were performed two times with three replicates in each experiment. Values represent means with SD. ***p ≤ 0.001. **e** HeLa cells seeded in 24-well plate were transfected with siNOP or siNeg (20 pmol each), and then infected with HSV-1/F at 0.1 MOI for the indicated times, and assessed as described in Fig. 3c. Values represent means of triplicates with SD. **p ≤ 0.01; ***p ≤ 0.001. **f** HeLa cells transfected with control plasmid (lanes 2, 4, 6, 8) or Flag-tagged N4 (lanes 3, 5, 7, 9) were mock-infected (lane 1) or infected with HSV-1/F at 10 MOI for indicated times (lanes 2–9). Cell lysates were then analyzed by Western blotting using antibodies directed against phospho-eIF2α (p-eIF2α), eIF2α, phospho-eIF4E (p-eIF4E), eIF4E, HSV-ICP8, Flag, and actin

Puromycin is an aminoacyl tRNA analog that is incorporated into the nascent chain C-terminus and terminates elongation. It has been used to directly monitor the translation rate of newly synthesized protein when used in minimal amounts^26^. To measure the effect of NOP53 on the translation rate upon HSV-1 infection, HeLa cells were transfected with Flag-tagged NOP53-N4 or negative control and then infected with HSV-1 at MOI of 1, and then subjected to pulse-chase puromycin labeling. In agreement with earlier findings^27^, gradual infection of HSV-1 moderately modulated global protein synthesis (Fig. 2a, lanes 2, 4, 6, 8). Furthermore, ectopic expression of NOP53 increased the accumulation of HSV-ICP8 (lanes 3, 5, 7, 9), but synthesis of cellular protein remained unaffected. This result suggests that NOP53 improves the translation of viral mRNA of HSV-1/F.

We then examined HSV-1 replication following over-expression or knockdown of NOP53. As shown in Fig. 2b, the ectopic expression of Flag-tagged NOP53 or NOP53-N4 resulted in an increase of viral yields by 86 and 31 fold, respectively, at 36 h.p.i., in comparison with the negative control, and an increase of viral yields by 60 and 42 fold at 48 h.p.i. RT-PCR was used to elucidate the levels of viral mRNA; we detected that ectopic expression of both NOP53 and NOP53-N4 significantly up-regulate the level of viral mRNA (Fig. 2c). NOP53-N4 also enhanced viral replication to a level comparable to that achieved under enhancement by wt NOP53 in HeLa cells. On the other hand, siRNA-mediated knockdown of NOP53 resulted in reduction of viral yields by 24 and 33 fold at 36 and 48 h.p.i., respectively (Fig. 2d), and also a reduction in the level of viral mRNA (Fig. 2e). These results indicate that NOP53 improves the production of viral particles and level of viral proteins of HSV-1/F, without affecting global protein synthesis.

### NOP53 regulates the activity of initiator trimeric eIF2 complex

Translation initiation requires the concerted activity of many cellular proteins, known as host eukaryotic initiation factors (eIFs). There are two main regulatory steps that control polypeptide chain initiation: the activity of the Met-tRNA-binding factor eIF2 complex and the formation of the eIF4F mRNA cap-binding complex^28,29^. The phosphorylation of eIF2α on serine 51 (Ser51) prevents recycling of the trimeric eIF2 complex, resulting in translation initiation attenuation^30,31^, while the eIF4E controls eIF4F complex formation and cellular translation via phosphorylation on serine 209 (Ser209)^32,33^. In this study, HeLa cells were transiently transfected with Flag-tagged NOP53-N4 or negative control and then infected with HSV-1 at MOI of 10 for 8, 12, 24, and 36 h. As shown in Fig. 2f, we found that HSV-1 infection significantly enhances the level of phospho-eIF2α (p-eIF2α) and phospho-eIF4E (p-eIF4E) at 8 and 12 h.p.i. (lanes 2, 4), but the phosphorylation levels gradually decreased as the infection progressed. Furthermore, the increase of NOP53-N4 greatly decreased p-eIF2α level, especially at 8 and 12 h.p.i. (lanes 3, 5), but did not alter the p-eIF4E level and the accumulation of total eIF2α or eIF4E at the indicated times. These results suggest that NOP53 regulates the activity of initiator trimeric eIF2 complex, rather than the formation of cap-eIF4F complex, to functionally promote the viral protein synthesis of HSV-1.

### NOP53 attenuates the phosphorylation level of endogenous and exogenous eIF2α

**Fig. 3:**
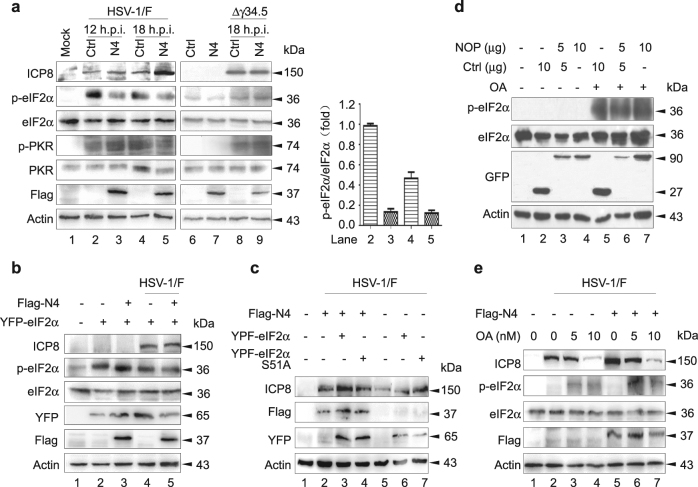
NOP53 is involved in dephosphorylation of eIF2α for HSV-1/F replication priority **a** HeLa cells transfected with control (lanes 2, 4, 6, 8) or Flag-tagged N4 (lanes 3, 5, 7, 9) were mock-infected (lanes 1, 6, 7) or either exposed to HSV-1/F (lanes 2–5) or HSV-1/Δγ34.5 (lanes 8–9) at 10 MOI for indicated times. Cell lysates were analyzed by Western blotting with specific antibodies against HSV-ICP8, p-eIF2α, eIF2α, phospho-PKR (p-PKR), PKR, Flag and actin. The change in abundance of p-eIF2α (lanes 2–5) was analysed by densitometric analysis using Image-Pro Plus Software and normalized to eIF2α (right). **b** HeLa cells were transfected with YFP-tagged eIF2α (lanes 2, 4) or co-transfected with Flag-tagged N4 (lanes 3, 5) for 36 h. The cells were then mock-infected (lanes 1–3) or infected with HSV-1/F at 10 MOI (lanes 4, 5) for 12 h, and cell lysates were analyzed by Western blotting with the indicated antibodies. One of two independent experiments is shown. **c** HeLa cells in T-25 flask were transfected with Flag-tagged N4 (lanes 2–4) or control plasmid (lanes 5-7) (5 μg each), along with YFP-tagged eIF2α (lanes 3, 6) or YFP-tagged eIF2αS51A (lanes 4, 7) (5 μg each), then mock-infected (lane 1) or infected with HSV-1/F at 10 MOI for 12 h (lanes 2–7). Cell lysates were then analyzed by Western blotting with the indicated antibodies. One of two independent experiments is shown. **d** HeLa cells seeded in T-25 flask were co-transfected with control plasmid (10, 5, 0 μg) and plasmid to express GFP-tagged NOP53 (0, 5, 10 μg) for 36 h. Cells were then treated with DMSO (lanes 1–4) or 10 nM of OA (lanes 5–7) for 9 h. Electrophoretically separated proteins were analyzed by Western blotting with the indicated antibodies. **e** HeLa cells transfected with control plasmid (lanes 2–4) or Flag-tagged N4 (lanes 5–7) were mock-infected (lane 1) or infected with HSV-1/F at 10 MOI for 12 h (lanes 2–7) in the absence or presence of OA. Cell lysates were analyzed by Western blotting with indicated antibodies

HSV-1 utilizes four proteins to counteract the activation of eIF2 kinases, in which Us11^34^, vhs^35^, and glycoprotein gB^36^ are able to block the activation of PKR or PKR-related endoplasmic reticulum kinase (PERK), while γ34.5 recruits cellular PP1α to dephosphorylate eIF2α^11–13^. The best-studied antiviral defense targeting viral translation is the dsRNA-dependent PKR^37^. To test whether NOP53 targets PKR for p-eIF2α depression, HeLa cells were transiently transfected with Flag-tagged NOP53-N4 or negative control, and then infected with HSV-1/F for 12 and 18 h. As shown in Fig. 3a, ectopic expression of NOP53-N4 resulted in a significant decrease in endogenous p-eIF2α of 10 and 5 fold at 12 and 18 h p.i. (lanes 3, 5), respectively, as compared with control cells (lanes 2, 4). The accumulation of PKR and its phosphorylation level, however, was relatively constant. It is inferred that NOP53 attenuation of p-eIF2α level is functionally correlated with viral protein γ34.5. We also found that the ectopic expression of NOP53-N4 fails to affect the phosphorylation levels of either PKR and eIF2α induced by infection with γ34.5 deletion mutant virus HSV-1/Δγ34.5. These results suggest that NOP53 is involved in γ34.5 dephosphorylation of eIF2α.

To further clarify whether NOP53 is responsible for eIF2α dephosphorylation, two constructs encoding the wt YFP-tagged eIF2α and the phosphorylation-incompetent variant eIF2α-S51A were transiently transfected or co-transfected with Flag-tagged NOP53-N4, followed by mock-infection or HSV-1/F infection. Consistent with previous reports^38^, ectopic expression of eIF2α improved p-eIF2α level (Fig. 3b, lane 2), while the increase in phosphorylation level remained unaffected when cells were co-transfected with NOP53-N4 (lane 3). There is no evidence supporting the idea that NOP53 directly targets eIF2α phosphorylation. We discovered that after HSV-1/F infection, the ectopic expression of NOP53-N4 leads to decreased accumulation of p-eIF2α, coincident with increased viral propagation (lane 5).

We further investigated whether NOP53-N4 regulating eIF2α activity is associated with HSV-1/F replication. As shown in Fig. 3c, ectopic expression of eIF2α-S51A (lane 7) but not wt eIF2α (lane 6) up-regulated the expression of viral ICP8, while ectopic co-expression of NOP53-N4 with eIF2α-S51A did not improve for further ICP8 accumulation (lane 4). Furthermore, ectopic co-expression of NOP53-N4 with wt eIF2α greatly promoted viral replication (lane 3), compared to either ectopic expression of NOP53-N4 (lane 2) or co-expression with eIF2α-S51A (lane 4). It is possible that NOP53 eliminates the sequestration of the trimeric eIF2 complex when wt eIF2α is over-expressed. Taken together, the results shown in Figs. 3a–c indicate that NOP53 attenuates the phosphorylation level of endogenous and exogenous eIF2α to counteract translational arrest and maintain HSV-1/F replication priority.

### PP1α is necessary for NOP53 attenuation of p-eIF2α

As indicated above, HSV-1 γ34.5 recruits phosphatase PP1α to reverse eIF2α-mediated translational arrest. It has been reported that PP1α contributes to HSV-1 pathogenesis and is required for disseminated disease in the brain^39^. To address whether NOP53 relates functionally with PP1α, okadaic acid (OA) was used to induce eIF2α phosphorylation in this study. OA is an inhibitor of the serine/theorine protein phosphatases PP1 and PP2A^40^. We found that the ectopic expression of NOP53 fails to affect the p-eIF2α induced by treatment with OA (Fig. 3d). After HSV-1/F infection, NOP53-N4 increase did not relieve p-eIF2α level, and no promotion of viral replication was observed (Fig. 3e). No evidence supports that NOP53 functionally mimics or regulates PP1α activity. Nevertheless, these results do demonstrate that PP1α is required for NOP53 attenuation of eIF2α phosphorylation.

### Cytoplasmic translocation of NOP53 is associated with HSV-1 replication

**Fig. 4:**
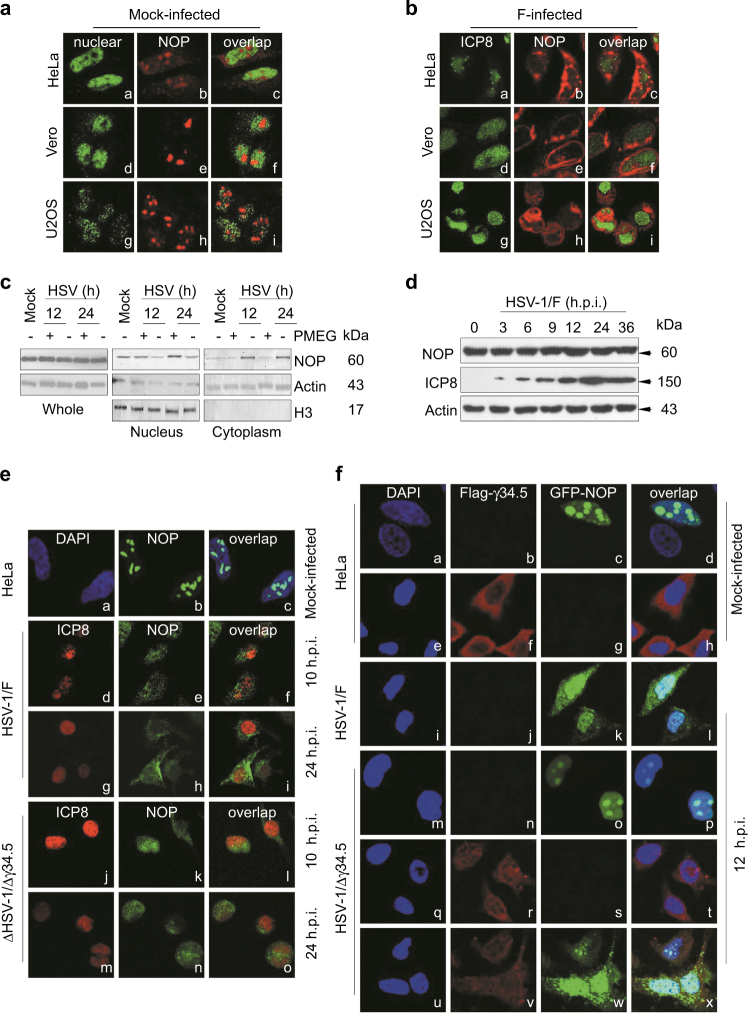
γ34.5 induces the cytoplasmic translocation of NOP53 **a, b** HeLa, Vero, or U2OS cells grown in 4 well slides were mock-infected **a** or exposed to 10 MOI of HSV-1/F **b**. At 12 h.p.i., cells were fixed and then reacted with antibodies to NOP53 (b, e, h), ICP8 (a, d, g for panel **b**), PDCD4 (a, d, g for panel **a**), and overlap (c, f, i). **c** HeLa cells were mock-infected or infected with HSV-1/F at 10 MOI for 12 or 24 h, in the presence of PMEG (20 μg/mL) or vehicle control DMSO. The cells were harvested and nuclei and cytoplasm were then isolated and analyzed by Western blotting with an antibody against NOP53. Actin and H3 were used as loading controls for separated cytoplasmic and nuclear proteins, respectively. **d** HeLa cells were infected with HSV-1/F at 1 MOI for 0, 3, 6, 9, 12, 24, or 36 h, cell lysates were prepared, and NOP53 and HSV-ICP8 were measured by Western blotting with specific antibodies, with actin as a control. **e** HeLa cells were mock-infected (a–c) or infected with HSV-1/F (d–i), or Δγ34.5 (j–o) at 10 MOI. Cells were fixed and then reacted with antibodies to NOP53 (b, e, h, k, n), HSV-ICP8 (d, g, j, m), and overlap (c, f, i, l, o). Nuclei were stained with DAPI (a). **f** HeLa cells were transfected with GFP-tagged NOP53 (c, k, o) or Flag-tagged γ34.5 (f, r), or co-transfected with Flag-tagged γ34.5 and GFP-tagged NOP53 (v, w). The cells were then mock-infected (a–h) or infected with either HSV-1/F (i–l) or Δγ34.5 (m–x) at 10 MOI. Cells were fixed and then reacted with antibodies to Flag (b, f, j, n, r, v), GFP (c, g, k, o, s, w), and overlap (d, h, l, p, t, x). Nuclei were stained with DAPI

To investigate the viral protein that is responsible for NOP53 function, we focused on the cellular localization of NOP53 after infection with HSV-1/F. We found that NOP53 is expressed as a set of discrete globular structures within the nuclei of different types of cell lines (Fig. 4a), consistent with previous work;^41^ then migrated from the nuclei upon infection at 12 h.p.i. by HSV-1/F (Fig. 4b). To clarify the relationship of NOP53 redistribution with viral replication, DNA polymerase inhibitor PMEG^42^ was used for nuclear and cytoplasmic separation analysis. As shown, replication inhibitor PMEG led to the sequestration of NOP53 in the nuclei of HSV-1/F infected cells, without affecting the level of NOP53 expression (Fig. 4c). To investigate whether viral infection might modulate NOP53 level in cells, we studied the time-course of NOP53 during HSV-1 infection, and detected that NOP53 level was not significantly affected (Fig. 4d). These results demonstrate that the endogenous level of NOP53 is sufficient for optimal viral replication. Accordingly, abrogating NOP53 redistribution diminished its ability to support HSV-1 replication.

### γ34.5 induces the redistribution of NOP53

We then explored whether HSV-1/F or Δγ34.5 infection leads to NOP53 migration as the infection progresses. As shown in Fig. 4e, endogenous NOP53 overwhelmingly migrated from nucleus to cytoplasm of HeLa cells after HSV-1/F infection at 12 and 24 h.p.i. (d-i); and the discrete globular structures were slightly dispersed in nuclei of HSV-1/Δγ34.5-infected cells (j-o). To further study whether γ34.5 participates in the redistribution of NOP53, we ectopically expressed GFP-tagged NOP53 in cells and then infected them with HSV-1/F (Fig. 4f, i–l) or HSV-1/ Δγ34.5 (m-p). The cytoplasmic translocation of exogenous NOP53 was observed only in HSV-1/F-infected cells. Upon infection, HSV-1/F exhibited nearly 100 fold higher replication than HSV-1/Δγ34.5;^43^ ectopic expression of γ34.5 can efficiently rescue viral replication of Δγ34.5 to the extent of HSV-1/F^13^. To verify whether γ34.5 directly stimulates NOP53 migration, HeLa cells were ectopically expressed with GFP-tagged NOP53 and Flag-tagged γ34.5 (u–x), and were then infected with HSV-1/Δγ34.5. Thus, the redistribution of exogenous NOP53 was observed in repaired HSV-1 (Δγ34.5 R) infected cells. These results demonstrate that γ34.5 induces the cytoplasmic translocation of NOP53.

### NOP53-derived recombinant protein attenuates p-eIF2α in cells infected with HSV-1/F

**Fig. 5:**
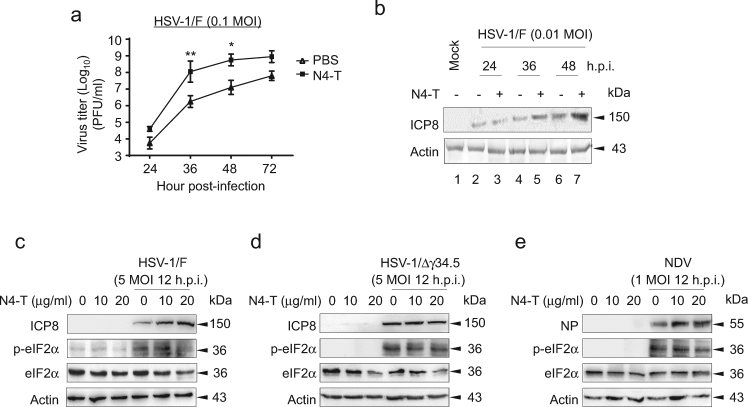
Recombinant protein N4-T attenuates p-eIF2α in the presence of γ34.5 **a** HeLa cells were exposed to HSV-1/F at 0.1 MOI for indicated times, in the presence of PBS or N4-T of 20 μg/ml. Viral yields were determined by plaque assay and were presented as log_10_ PFU/ml. Values represent means of triplicates with SD. *p ≤ 0.05; **p ≤ 0.01. **b** HeLa cells were infected with HSV-1/F at 0.01 MOI in the presence of PBS or N4-T of 20 μg/ml. Expression of HSV-ICP8 was detected by Western blotting at 24, 36, or 48 h.p.i., and actin served as the loading control. **c-e** HeLa cells infected with HSV-1/F **c**, HSV-1/Δγ34.5 **d**, or NDV **e** were treated with N4-T at different concentrations of 0, 10, or 20 μg/ml. Cell lysates prepared 12 h.p.i. were then analyzed by Western blotting with the indicated antibodies. Shown is one representative of two independent experiments

Our previous study revealed that NOP53-derived recombinant protein N4-T, forming α-helical dimer, promotes replication of seven different viruses via suppression of the IFN-β antiviral response^44^. In consideration of the relationship between NOP53 redistribution and viral replication priority, N4-T protein was employed in this study. First, HeLa cells were exposed to HSV-1/F in the presence or absence of N4-T, to explore whether N4-T promotes viral proliferation. As shown in Fig. 5a, the viral yields of HSV-1/F at 24, 36, 48, and 72 h.p.i. were 6, 62, 44, and 13 fold higher, respectively, in cells treated with N4-T at a concentration of 20 μg/ml, than in cultures treated with PBS. We also explored the effect of N4-T on the level of viral protein throughout the infection course, and found significantly up-regulated expression of HSV-ICP8 (Fig. 5b). We further examined whether N4-T promotes HSV-1 replication via regulation of eIF2α phosphorylation. The results revealed that p-eIF2α level is gradually decreased and HSV-ICP8 expression is up-regulated, with increasing concentrations of N4-T (Fig. 5c). N4-T treatment failed, however, to affect the levels of p-eIF2α and HSV-ICP8 upon HSV-1/Δγ34.5 infection (Fig. 5d). N4-T also had no effect on the p-eIF2α level induced by unrelated Newcastle disease virus (NDV) infection, although it up-regulated the expression of viral protein NP (Fig. 5e). It is possible that N4-T promotes NDV replication by suppressing the activity of IFN-β. These findings indicate that cytoplasmic NOP53 directly depresses p-eIF2α level in the presence of γ34.5.

### NOP53 has a mild effect on the replication of HSV-1/Δγ34.5 in Vero cells

**Fig. 6:**
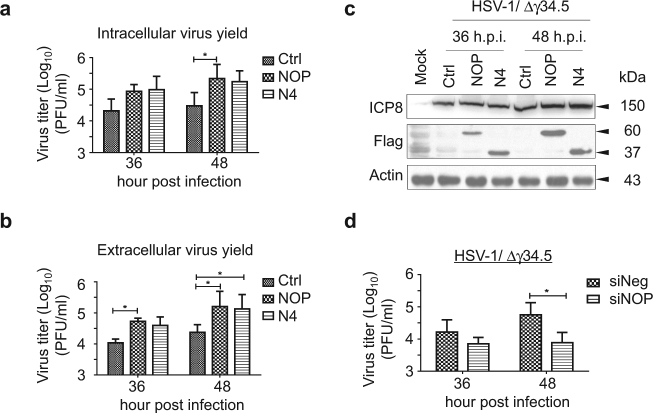
NOP53 subtly promotes the replication of HSV-1/Δγ34.5 in Vero cells **a, b** Vero cells were transfected with control plasmid or plasmids to express Flag-tagged NOP53 or Flag-tagged N4. The cells were then infected with HSV-1/Δγ34.5 at 0.1 MOI. Intracellular **a** or extracellular **b** viral yields were determined 36 or 48 h.p.i., and then assessed as described in Figs. 1a and 1b. Values represent means of triplicates with SD. *p ≤ 0.05. **c** Vero cells were transfected with control plasmid or plasmids to express Flag-tagged NOP53 or Flag-tagged N4, and then infected with HSV-1/Δγ34.5 at 0.1 MOI. Cell lysates prepared 36 or 48 h.p.i. were then analyzed by Western blotting using antibodies directed against HSV-ICP8, Flag and actin. **d** Vero cells transfected with siNOP or siNeg were infected with HSV-1/Δγ34.5 at 0.1 MOI, and then assessed as described in Fig. 1d. Values represent means of triplicates with SD. *p ≤ 0.05

In the current study, we addressed the effect of over-expression or knockdown of NOP53 on intracellular and extracellular viral yields in HSV-1/Δγ34.5 infected Vero cells (Fig. 6). We found that the ectopic expression of wt NOP53 or NOP53-N4 followed by infection with HSV-1/Δγ34.5 moderately raises intracellular viral yields by 4.9 and 3.6 fold respectively at 36 h.p.i., and by 6.2 and 4.8 fold respectively at 48 h.p.i., in comparison with negative control (Fig. 6a). Increase of NOP53 and NOP53-N4 resulted in an increase in extracellular viral yields of 3.9 and 2.6 fold respectively at 36 h.p.i., and 5.8 and 4.7 fold respectively at 48 h.p.i. (Fig. 6b). Under the current experimental conditions, we found that both NOP53 and NOP53-N4 subtly promote viral replication, as indexed by viral protein HSV-ICP8 (Fig. 6c). On the other hand, knockdown of NOP53 barely reduced the production of viral particles. At 36 and 48 h.p.i., NOP53 decrease resulted in reduction of viral yields by 1.3 and 6.3 fold respectively (Fig. 6d). These results reveal that NOP53 has a mild effect on the replication of HSV-1/Δγ34.5 in Vero cells, indicating that γ34.5 is required for NOP53 function to promote HSV-1 replication in type I IFN-independent manner.

### NOP53 interacts directly with γ34.5

**Fig. 7:**
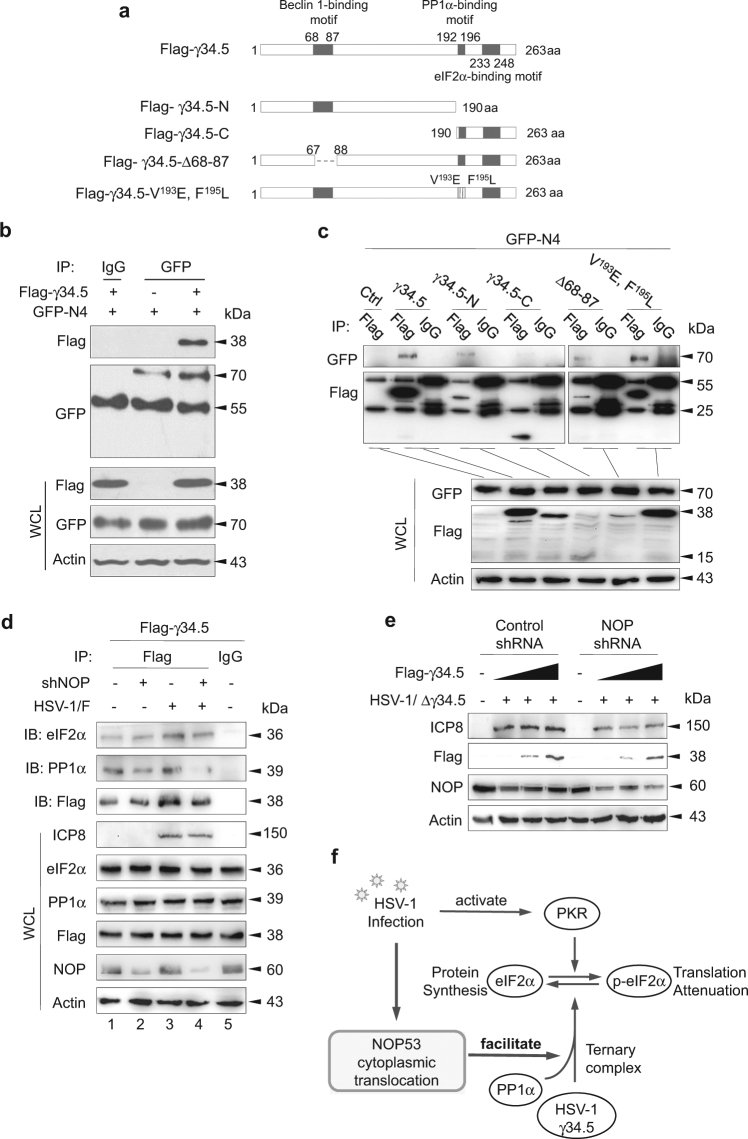
NOP53 is essential for γ34.5 recruitment of PP1α **a** Schematics of Flag-tagged γ34.5 mutant constructs. **b** HEK293T cells in T-25 flask were transfected with plasmid to express GFP-tagged N4 or co-transfected with plasmids to express GFP-tagged N4 and Flag-tagged γ34.5 for 36 h (5 μg each). Cell lysates were immuno-precipitated with mouse anti-GFP or mouse IgG antibody and subjected to Western blotting with specific antibodies to detect Flag or GFP. Whole cell lysates (WCL) were also examined to confirm the expression of proteins from the transfected plasmids. **c** HEK293T cells were co-transfected with plasmids to express GFP-tagged N4 and Flag-tagged γ34.5, γ34.5 variants indicated in **a**. Cell lysates were immuno-precipitated with mouse anti-Flag or mouse IgG antibody and subjected to Western blotting with specific antibodies to detect GFP or Flag. **d** HeLa cells in T-25 flask were transfected with control shRNA (lanes 1, 3) or NOP53-specific shRNA (lanes 2, 4) for 24 h, followed by transfection of Flag-tagged γ34.5 for 24 h, then infected with HSV-1/F at 10 MOI for 12 h. Cell lysates were immuno-precipitated with anti-Flag or mouse IgG antibody and subjected to Western blotting with specific antibodies to detect eIF2α, PP1α, or Flag. **e** HeLa cells were transfected with control shRNA or NOP53-specific shRNA followed by transfection of Flag-tagged γ34.5 (0, 5, 10 μg), then infected with HSV-1/Δγ34.5 at 0.1 MOI. Cell lysates prepared 36 h.p.i. were then analyzed by Western blotting using antibodies directed against HSV-ICP8, Flag, NOP53, and actin. **f** Schematic illustration of the mechanism

Previous reports showed that γ34.5 is related to various cellular proteins, and it has been shown to contain a Beclin-1 binding motif at amino acids 68–87, a PP1α binding motif at amino acids 192-196, and an eIF2α binding motif at amino acids 233–248^10,13^. The crucial functional regions of γ34.5 that are associated with NOP53 were determined by construction of Flag-tagged γ34.5 and a series of variant plasmids, including N-terminus region (residues 1 to 190), C-terminus region (residues 190 to 263), Beclin-1 binding motif deleted mutant (Δ68–87), and site mutation of V^193^E and F^195^L (Fig. 7a). Specifically, both the V^193^ and F^195^ sites are required for γ34.5 to bind to PP1α^13^. In the present study, we used GFP-specific antibody to immune-precipitate ectopically-expressed GFP-tagged NOP53-N4, and used Flag-specific antibody in Western blotting to detect whether Flag-tagged γ34.5 was present in the immune complex. The result revealed that NOP53-N4 can interact with γ34.5 (Fig. 7b). Furthermore, we found that NOP53-N4 is in the immune complex with γ34.5 and its variants; no interaction was observed, however, between NOP53-N4 and the C-terminus region of γ34.5 (Fig. 7c). To summarize, NOP53 interacts directly with γ34.5 but does not rely on γ34.5 binding to cellular proteins PP1α, Beclin-1, or eIF2α.

### NOP53 is essential for γ34.5 recruitment of PP1α to maintain HSV-1 virulence

We then explored whether NOP53 knockdown affects the interaction between γ34.5 and PP1α or eIF2α. As shown in Fig. 7d, the interaction of γ34.5 and PP1α was significantly disrupted in NOP53 knockdown cells, regardless of viral infection, but the interaction of γ34.5 and eIF2α remained unaffected. This result demonstrated that NOP53 is responsible for γ34.5 recruitment of PP1α. In order to further understand the effect of NOP53 decrease on the function of HSV-1 γ34.5 as a virulence factor, HeLa cells were transiently transfected with NOP53-specific shRNA or negative control, then transfected with increasing doses of Flag-tagged γ34.5, and then infected with HSV-1/Δγ34.5. As shown in Fig. 7e, the ectopic expression of γ34.5 up-regulated the expression of HSV-ICP8 in control cells, but failed to play a role in NOP53 knockdown cells. Our findings revealed that NOP53 is essential for γ34.5 to maintain HSV-1 virulence. We also found that NOP53 knockdown does not repress the accumulation of exogenous γ34.5, demonstrating that NOP53 is unrelated to regulating the stabilization of γ34.5. To summarize (Fig. 7f): γ34.5 induces NOP53 redistribution, and the cytoplasmic NOP53 facilitates γ34.5 recruitment of PP1α to dephosphorylate eIF2α for optimal viral replication.

### NOP53 knockdown reduces HSV-1/F growth and pathogenesis in mice

**Fig. 8:**
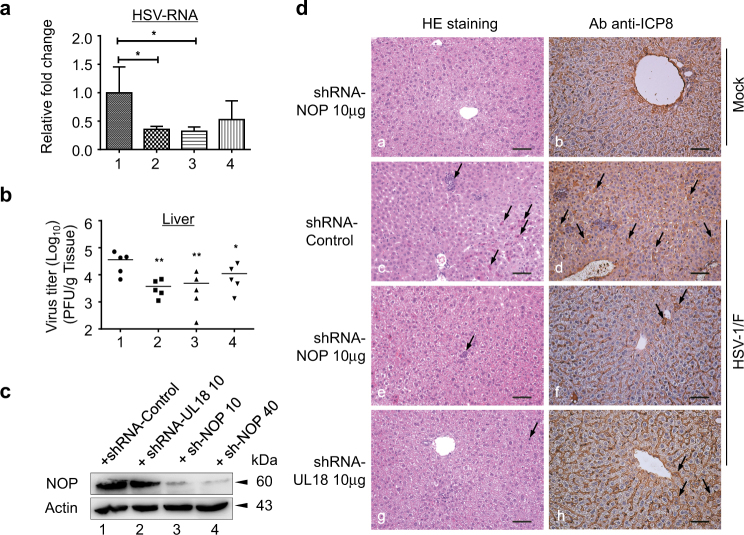
NOP53 knockdown reduces HSV-1/F growth and pathogenesis in mice **a** BALB/c mice were injected i.p. with control shRNA (lane 1), UL18-specific shRNA (lane 2), NOP53-specific shRNA (lane 3) (10 μg each), or NOP53-specific shRNA (40 μg, lane 4) for 24 h. The mice were then infected with HSV-1/F (10^7^ PFU/ mice). Mice were killed 5 days later and the livers were harvested, total RNAs were isolated. Expression levels of HSV-ICP8 mRNA were quantified by qRT-PCR and normalized with GAPDH. Values represent means of triplicates with SD. *p < 0.05. **b** Under the same experimental condition, mice livers (*n* = 5) were homogenized in media and quantified for viral yields by plaque assay, and were presented as log_10_ PFU/ml. Data are representative of two independent experiments, *p < 0.05; **p < 0.01. **c** Efficiency of knockdown was analyzed by Western blotting with specific antibody to NOP53, with actin as a control. **d** Representative images of H&E staining (left) and HSV-ICP8 (right) in liver from mice infected with HSV-1/F. Arrows in left panel indicate eosinophilic, inflammatory cell infiltration, necrotic cells with pyknotic nuclei. Scale bars, 50 μm

To investigate the effect of NOP53 on HSV-1/F infection *in vivo*, NOP53-specific shRNA was administered to BALB/c mice with the aid of *in vivo*-jet PEI. The mice were then infected with HSV-1 and the livers were harvested 5 days post infection. Consistent with a previous report^45^, the knockdown of capsid protein VP23 (UL18) suppressed the level of viral mRNA (Fig. 8a, lane 2). Our results also revealed that 10 μg of NOP53-specific shRNA treatment 24 h prior to infection with HSV-1/F reduces viral mRNA level in livers (lane 3), whereas 40 μg had only a slight effect (lane 4). Consistently, treatment of mice with 10 μg of NOP53-specific shRNA or UL18-specific shRNA significantly decreased viral yields, by 10 and 13 fold respectively, compared with control treated mice (Fig. 8b). In addition, both 10 μg and 40 μg of NOP53-specific shRNA were able to suppress the expression of NOP53 in mice (Fig. 8c). Our results indicate that NOP53 depression inhibits efficient viral replication of HSV-1 in mice.

To determine the ability of NOP53-specific shRNA to protect against hepatic injury after viral infection, the mice were injected with different shRNAs and then infected with HSV-1/F. Using immunohistochemistry (IHC) staining, we examined the pathological changes and the distribution of HSV-1 in the livers (Fig. 8d). Eosinophils, inflammatory cell infiltration, and necrotic cells with pyknotic nuclei were observed in the livers of infected mice (**c**). In NOP53 knockdown mice (**e**) and UL18 knockdown mice (**g**), no obvious inflammatory cell infiltration was observed, although a few eosinophils and necrotic cells were observed. Furthermore, there was a large number of ICP8-positive cells in the livers of control shRNA treated mice (**d**); the numbers were greatly decreased under treatment with NOP53-specific shRNA (**f**) or UL18-specific shRNA (**h)**. The results indicate that NOP53 depression significantly reduces both virus growth *in vivo* and the associated inflammatory response.

## Discussion

Host cell-dependent viral translation is a crucial step for viral replication. The phosphorylation of eIF2α plays a principal role in shutoff of protein translation to protect host cells against viruses. Therefore, many viruses have evolved mechanisms to evade or subvert this antiviral defense response for successful replication. Previous studies have demonstrated that certain viral gene products are able to recruit PP1α to counteract eIF2α phosphorylation; these include the DNA virus protein DP71L protein of African swine fever virus (ASFV)^46,47^, E6 oncoprotein of human papillomavirus (HPV)^48^, and RNA virus protein 7 of transmissible gastroenteritis virus (TGEV)^49^. It is well understood that HSV-1 γ34.5 mediates the dephosphorylation of eIF2α, but the cellular regulatory protein that is involved in this process has yet to be determined.

Here we provide evidence that an essential virulence factor, γ34.5, induces cytoplasmic translocation of NOP53, which facilitates γ34.5 recruitment of PP1α to dephosphorylate eIF2α. This is the first report of cellular regulatory protein NOP53 being required for efficient viral replication of HSV-1. Understanding this mechanism not only provides important information on the process of viral replication, but will also be useful for developing potential target inhibitors against infection.

HSV-1 infection triggers a rapid induction of host innate immune responses. Herpesvirus DNA can be recognized intracellularly by a variety of DNA sensors, with the best documented being the stimulator of interferon genes (STING) pathway, which initiates subsequent type I IFN production^50,51^. It has recently been reported that RIG-I-mediated STING up-regulation restricts HSV-1 infection^52^. Our previous study revealed that NOP53 targets RNA sensor RIG-I to suppress the activity of IFN-β for efficient viral replication of vesicular stomatitis virus (VSV) and NDV^21^. It has been reported that HSV-1/Δγ34.5 infection produces greater amounts of IFN-β than in HSV-1/F-infected cells^53^. We found, however, that the increase of NOP53, in two forms of transient transfection and *in vitro* expression, was unable to dephosphorylate eIF2α and promote viral replication upon HSV-1/Δγ34.5 infection. In addition, NOP53 promoted HSV-1/F replication in type I IFN-deficient Vero cells. Two major lines of evidence thus support that γ34.5 manipulates NOP53 to promote HSV-1 replication mainly by regulating IFN-independent eIF2α activity. This improves our understanding of how the viruses manipulate NOP53 for efficient replication via negatively regulating distinct antiviral responses produced by the infected cells.

Growth arrest and DNA damage-inducing protein 34 (GADD34), an eIF2α phosphatase, recruits protein PP1α to dephosphorylate p-eIF2α in unfolded protein response (UPR)^54^. Viral infections sometimes up-regulate the expression of GADD34^55–57^. The C-terminal domain of γ34.5 is partially homologous to the corresponding domains of protein encoded by GADD34^58^. It remains unknown whether NOP53 is involved in GADD34 recruitment of PP1α in response to viral infection. Notably, NOP53 is able to interact with different kinds of viral proteins (ICP0, ICP22^41^, γ34.5) and cellular proteins (p53, RIG-I, PP1α). Understanding the impact of NOP53 conformation change and corresponding function upon viral infections is a promising direction for future work.

## Materials and methods

### Cells, viruses, and drugs

Vero, HeLa, U2OS and HEK293T cells obtained from ATCC were maintained in Dulbecco’s minimal Eagle’s medium (DMEM; Invitrogen, Carlsbad, CA, USA) supplemented with 10% fetal bovine serum (FBS; Gibco, Grand Island, NY, USA), 100 μg/ml penicillin, and 100 μg/ml streptomycin. All cells were cultured at 37 °C in a humidified atmosphere containing 5% CO_2_. The wild-type virus HSV-1(F) and Δγ34.5 were kindly provided by Bernard Roizman. Puromycin was purchased from Merck Millipore (Billerica, MA, USA) and used at a final concentration of 10 μg/ml. 9-[2-(phosphonomethoxy) ethyl] guanine (PMEG) was purchased from Sigma-Aldrich (St. Louis, MO, USA) and used at a final concentration of 20 μM. Okadaic acid (OA, Beyotime Biotechnology, Jiangsu, China) was used at a final concentration of 10 nM.

### Antibodies

Mouse monoclonal anti-ICP8 (sc-53329), mouse monoclonal anti-PDCD4 (sc-376430), and rabbit polyclonal anti-H3 (sc-10809) were purchased from Santa Cruz Biotechnology (Santa Cruz, CA, USA). Rabbit polyclonal anti-eIF2α (9722), rabbit monoclonal anti-phospho-eIF2α (Ser 51) (3398), rabbit polyclonal anti-phospho-eIF4E (Ser 209) (9741), rabbit polyclonal anti-PKR (3072), and rabbit polyclonal anti-phospho-PKR (Thr 446) (3076) were purchased from Cell Signaling Technology (Danvers, MA, USA). Rabbit polyclonal against NOP53 (ab131002) was purchased from Abcam (Cambridge, MA, USA). mAb 12D10 against puromycin was purchased from Merck Millipore. Rabbit polyclonal to eIF4E (11149-1-AP) was purchased from proteintech (Wuhan, China). Monoclonal ANTI-FLAG® M2 antibody produced in mouse (F1804) was purchased from Sigma-Aldrich. Mouse monoclonal anti-Actin (AA128), mouse monoclonal anti-GFP (AG281), goat anti-mouse secondary antibodies conjugated to horseradish peroxidase (HRP) (A0216), goat anti-rabbit secondary antibodies conjugated to HRP (A0208) used for Western blotting were all purchased from Beyotime Biotechnology. Goat anti-rabbit IgG (H + L) secondary antibody conjugated to Alexa Flour 594 (R37117) and goat anti-mouse IgG (H + L) secondary antibody conjugated to Alexa Flour 488 (R37120) used for immunofluorescence were purchased from Invitrogen.

### Plasmids

The vectors pFlag-CMV3 and pEGFP-N1 were purchased from Clontech (Mountain View, CA, USA). NOP53 and truncated variant N4, HSV-1 γ34.5 and γ34.5 mutants were cloned in vector using specific primers. Eukaryotic expression plasmids containing the genes were transfected into cells with the aid of Lipofectamine^TM^ 2000 (Invitrogen, Carlsbad, CA, USA) according to the manufacturer’s instructions.

### RNA interference *in vitro*

Cells were transfected with siRNA targeting NOP53 (Santa), or with pGMLV-SC1 vector encoding NOP53-specific shRNA, by means of Lipofectamine^TM^ 2000 (Invitrogen) according to manufacturer’s protocols. The shRNA target sequence used in this paper was 5′-GCT GAC AAA GAA GAG AAC CAA TTC AAG AGA TTG GTT CTC TTC TTT GTC AGC TTT TTC CAT GG-3′.

### MTT assay

Cell viability was assessed using a CellTiter 96® Non-Radioactive Cell Proliferation Assay (MTT) (Promega, Madison, USA) according to the manufacturer’s instructions. All experiments were performed in triplicate.

### Plaque formation assay

Vero cells seeded at 2.5 × 10^5^ cells per well in standard 12-well plates were infected with virus for 1.5 h and resuspended in DMED containing 1% FBS and 0.5% low-melt point agarose in DMEM for 72 h. The agarose was removed by suction and stained with 0.05% crystal violet in ethanol. Experiments were performed in triplicate. The results represent the averages from at least three independent experiments. Error bars indicate the standard deviation of the mean.

### Preparation of cell lysates and Western blotting

The cells were harvested at times indicated in the Results Section by scraping, collected by centrifugation, rinsed with phosphate-buffered saline (PBS) containing protease inhibitor cocktail and phosphatase activity inhibitor PhosSTOP (Roche, Basel, Switzerland), dissolved in 200 μl lysis buffer (pH7.5 20 mM Tris-HCl, 150 mM NaCl, 1% Triton X-100, 1 mM EDTA, 2.5 mM Sodium pyrophosphate, 1 mM β-Glycerrophosphate, 1 mM Sodium orthovanadate, 1 μg/ml Leupeptin) in the presence of the inhibitor and finally disrupted by sonication. Solubilization of protein harvested from cells, electrophoresis in denaturing polyacrylamide gels transferred to nitrocellulose serum, pretreatment with 5% milk, and reaction with appropriate antibodies were done. The protein bands were detected with secondary antibodies conjugated to HRP, where actin served as a loading control. In some parts of the work, histone H3 served as a loading control for separated nuclear proteins.

### Quantitative real-time PCR (RT-PCR)

Replicate cultures were harvested and total RNA was extracted with Trizol (Invitrogen). Total RNA was reverse transcribed into cDNA using a reverse transcription system (Promega). A two-step RT-PCR (SYBR Green I technology, Applied Roche) was performed using SYBR green supermix (Toyobo, Shanghai, China) according to the manufacturer’s protocol to measure transcription levels for several genes of interest. The primers used are as follows. GAPDH: 5′-CTG GTG ACC CGT GCT GCT T-3′ (forward), 5′-TTG CCG CCT TCT GCC TTA-3′ (reverse). ICP8: 5′-GACGGGCAATCAGCGGTTCG-3′ (forward), 5′-TCGTCCAGGTCGTCGTCATCC-3′ (reverse).

### Puromycin labeling

HeLa cells were infected with HSV-1/F and then labeled with 10 μg/ml puromycin for 1 h at different time points. After puromycin labeling, all cells were washed three times in cold PBS and lysed. Equivalent amounts of protein were separated by 12% SDS-PAGE, and subjected to Western blotting analysis using anti-puromycin antibody or indicated antibodies.

### Confocal microscopy analysis

Cells growing on glass coverslips were fixed with 4% paraformaldehyde for 15 min, quenched with 100 mM glycine for 15 min, permeabilized with 0.1% Triton X-100 for 15 min, and blocked with PBS containing 10% goat serum plus 1% BSA for 2 h. The cells were washed three times with PBS and incubated with diluted rabbit anti-NOP53, mouse anti-Flag, or mouse anti-ICP8 antibodies overnight at 4 °C. Cells were washed with PBS three times at room temperature, then incubated with fluorochrome-conjugated secondary antibodies in the dark for 1 h at room temperature. The cells were then rinsed and incubated with DAPI (Beyotime Biotechnology) for 5 min, rinsed and mounted. The cells were examined and images were captured using 100× objectives with an Olympus FluoView™ FV1000. The images were processed using FV10-ASW 4.0 software.

### Nuclear and cytoplasmic separation

HeLa cells were mock-infected or exposed to HSV-1/F at the MOI of 10. Cytoplasm and nuclei were then isolated using a professional kit (Pierce, Rockford, IL, USA, No. 78833) and analyzed by Western blotting with appropriate primary and secondary antibodies.

### Immunoprecipitation

Cells were washed 3 times with PBS and resuspended in immunoprecipitation buffer (Beyotime Biotechnology) in the presence of a protease inhibitor cocktail, then were disrupted by sonication, and clarified by centrifugation at 6000 rpm for 10 min. Cell lysates (0.5 mg) were precleared with 20 μl of protein A/G-Sepharose beads (Santa Cruz) for 60 min. Nonspecific complexes were pelleted by centrifugation at 10,000×*g* at 4 °C for 10 min. The supernatants were removed and incubated with either 2.5 μg of anti-Flag antibody, anti-GFP antibody, or the isotype control IgG at 4 °C for 12 h before the addition of 20 μl of protein A/G-Sepharose beads, and incubated for another 4 h in an end-over-end rotor. The immunoprecipitates were pelleted and washed five times with a lysis buffer. After the final wash, the pellet was resuspended in 40 μl of 2× SDS-PAGE loading buffer and boiled for 10 min before being analyzed by Western blotting with appropriate primary and secondary antibodies.

### Protein expression and purification

N4-T, in which Tat (GGSRYGRKKRRQRRR) was conjugated at the C-terminal of N4, was expressed and purified as described previously^44^. Cells treated with N4-T were harvested for plaque formation assay or Western blotting analysis.

### Transfection of shRNA and infection of HSV-1 *in vivo*

BALB/c mice (6–8 weeks old) were obtained from Beijing Vital River Laboratory Animal Technology Co., Ltd. Invitrogen BLOCK-iT^TM^ RNAi (https://rnaidesigner.thermofisher.com) was used to design shRNA targeting murine NOP53. Based on shRNA screen results, the shRNA target sequence used in this paper was 5′- GCCTTTGATTGGTCAGGATGC-3′. The shRNA above were injected intravenous (i.v.) with the aid of *in vivo*-jet PEI (Polyplus transfection, New York, NY, USA) 24 h before the mice were exposed to HSV-1/F (*n* = 5). The mice were infected intraperitoneal (i.p.) with HSV-1 (10^7^ PFU/mice) and were killed 5 days post infection and the livers were harvested, homogenized in media and quantified for viral yields by standard plaque assay. Viral protein ICP8 mRNA level was detected by RT-PCR. All animal protocols were approved by the Animal Welfare Committee of China Agricultural University and housed with pathogen-free food and water under 12 h light-cycle conditions.

### Immunohistochemical staining

The mice indicated above were sacrificed at the indicated days post infection, and then livers were harvested and fixed in 10% neutral buffered formalin. Organs were then paraffin-embedded, sectioned, and stained with hematoxylin and eosin and subjected to immunohistochemical analysis to HSV-ICP8 (diluted at 1:200). Pathological changes were observed under an Olympus microscope (BX41; Olympus, Tokyo, Japan).

### Statistics

All results are expressed as the means and standard deviations (SD). Statistical analyses were performed using Prism 5.01 (GraphPad Software). Significance was determined by two-way ANOVA followed by Tukey’s multiple comparisons test.

## Supplementary information

Supplementary Figure 1 Overexpression or knockdown of NOP53 did not affect the cell viability
