# Supplementary material for: Multifunctional viral protein γ34.5 manipulates nucleolar protein NOP53 for optimal viral replication of HSV-1
Source: Cell Death Dis. 2018 Jan 24;9(2):103. doi: 10.1038/s41419-017-0116-2 (PMC5833762; doi:10.1038/s41419-017-0116-2)
Supplement: Supplementary file 1 — Supplementary Figure 1 Overexpression or knockdown of NOP53 did not affect the cell viability [file 41419_2017_116_MOESM1_ESM.doc]

**Supplementary Information**

**
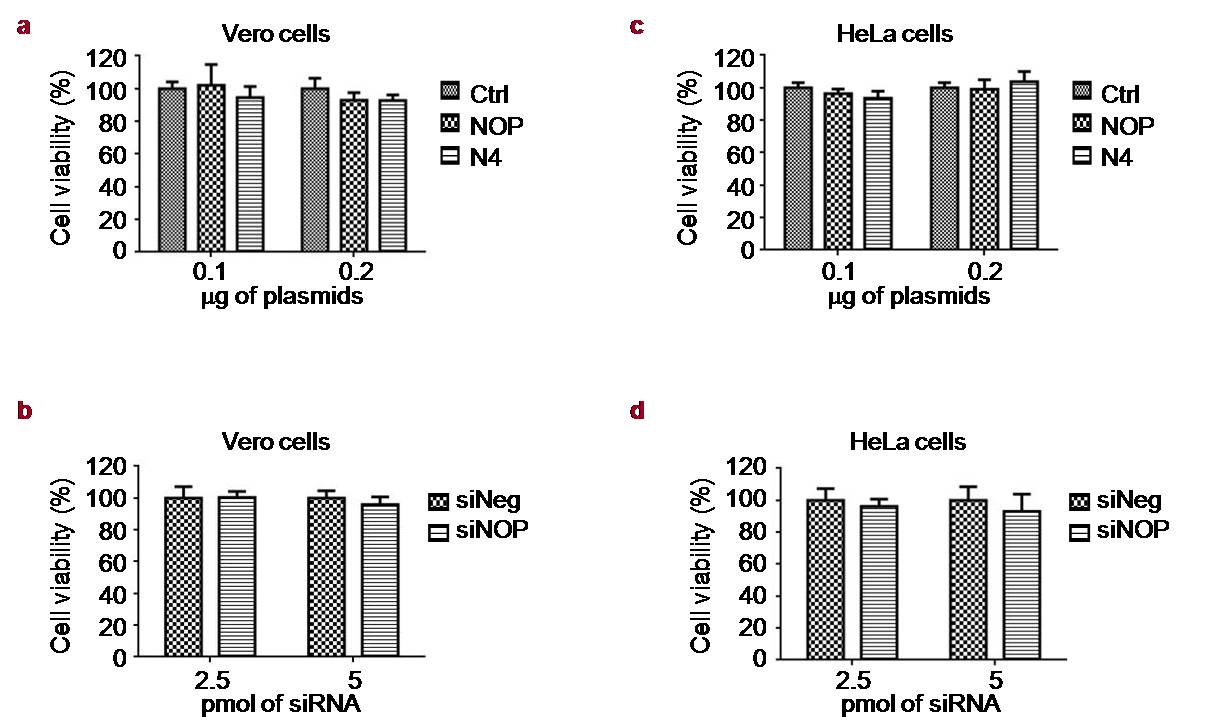
**

**Supplementary Figure 1 Overexpression or knockdown of NOP53 did not affect the cell viability. V**ero **(a)** or HeLa **(c)** cells seeded in 96-well plates were transfected with control plasmid or plasmids to express Flag-tagged NOP53 or Flag-tagged N4. Vero **(b)** or HeLa **(d)** cells were transfected with specific siRNA targeting NOP53 (siNOP) or negative siRNA (siNeg) for 72 h. The cell viability was analyzed by MTT assay. Values represent means of triplicates with standard deviations.
